# Comparison of calculation methods for estimating annual carbon stock change in German forests under forest management in the German greenhouse gas inventory

**DOI:** 10.1186/s13021-016-0053-x

**Published:** 2016-06-22

**Authors:** Steffi Röhling, Karsten Dunger, Gerald Kändler, Susann Klatt, Thomas Riedel, Wolfgang Stümer, Johannes Brötz

**Affiliations:** 1 Thünen Institute of Forest Ecosystems, Alfred-Möller-Straße 1, 16225 Eberswalde, Germany; 2 Forstliche Versuchs-und Forschungsanstalt Baden-Württemberg, Wohnhaldestraße 4, 79100 Freiburg, Germany

**Keywords:** Greenhouse gases, CO_2_, Carbon stock, National greenhouse gas inventory, Above ground biomass, Below ground biomass, National forest inventory, Stock-difference method, Emission factor

## Abstract

**Background:**

The German greenhouse gas inventory in the land use change sector strongly depends on national forest inventory data. As these data were collected periodically 1987, 2002, 2008 and 2012, the time series on emissions show several “jumps” due to biomass stock change, especially between 2001 and 2002 and between 2007 and 2008 while within the periods the emissions seem to be constant due to the application of periodical average emission factors. This does not reflect inter-annual variability in the time series, which would be assumed as the drivers for the carbon stock changes fluctuate between the years. Therefore additional data, which is available on annual basis, should be introduced into the calculations of the emissions inventories in order to get more plausible time series.

**Results:**

This article explores the possibility of introducing an annual rather than periodical approach to calculating emission factors with the given data and thus smoothing the trajectory of time series for emissions from forest biomass. Two approaches are introduced to estimate annual changes derived from periodic data: the so-called logging factor method and the growth factor method. The logging factor method incorporates annual logging data to project annual values from periodic values. This is less complex to implement than the growth factor method, which additionally adds growth data into the calculations.

**Conclusion:**

Calculation of the input variables is based on sound statistical methodologies and periodically collected data that cannot be altered. Thus a discontinuous trajectory of the emissions over time remains, even after the adjustments. It is intended to adopt this approach in the German greenhouse gas reporting in order to meet the request for annually adjusted values.

## Background

National emissions stemming from anthropogenic activities and their alternating trends in various sectors and times shall be estimated to improve the understanding of ongoing global greenhouse gas (GHG) fluxes as stated in the framework convention on climate change (Articles 4 and 12 of UNFCCC, 1992 [1]) and reiterated in several documents since then, such as the recent “Paris Agreement” [2]. Estimation of GHG emission and removal patterns and their changes over time enables decision makers in government and private industry to develop future action plans and policies towards mitigation of emissions. Therefore as well as for various other reasons GHG inventories are implemented to estimate emissions and removals [3–6]. Information on trends of emissions and removals are used e.g. as data provision for scientific models, for tracking progress of policy implementation and establishment of emissions compliance standards by regulatory agencies.

During the development of the United Nations Framework Convention on Climate Change (UNFCCC), which aims to “stabilize the global GHG concentration in the atmosphere at a level that would prevent dangerous human-induced interference with the climate system” [1] in [7] a system was created for transparently reporting of anthropogenic GHG emissions and removals which also mentioned in decision 24/CP.19 of the 19th conference of the parties under the UNFCCC [8]. The reporting according to this is following an specific reporting guideline framework elaborated by the Intergovernmental Panel on Climate Change (IPCC) [5, 9, 10] based on literature and good practices developed by technical experts [11–13].

Since plant growth reflects the possibility of removing CO_2_ from the atmosphere especially the plant growth rate, or increment, influences the performance of forest (wooded) ecosystems to uptake CO_2_. On the other hand, emissions are caused by biomass losses (harvest, disturbance and mortality). The combination of these two opposite effects results in net emissions or removals of CO_2_ which are also expressed as carbon stock changes. Calculations on this within the preparation of the German inventory on CO_2_ balances of forests are based on available data from National forest inventories which are carried out periodically and therefore do until now only deliver average values for time periods. As the times series of the inventories are also used to assess impacts of policies and management changes over time the following research focuses on methods for improvement of the GHG inventory and thus prepare a more thorough basis for decision making. This article attempts to introduce an approach for an annual estimation of carbon stock change which extends the actually used calculation method on stock changes by additionally incorporating harvest statistics and information on increment available for Germany.

With the use of the methods presented, instead of periodic values annual ones can be estimated in order to reflect inter-annual variation of wood harvest and increment in German forests and their influences on the emission factors.

## Methods

### Determination of biomass carbon stocks using forest inventory data

#### Forest inventory data

National forest inventories (NFI) are the primary source of forest information and are recognized as an important data source for estimating forest carbon stocks [14]. The NFI has been performed in Germany three times so far and was conducted in the periods between 1986–1988 (NFI 1987), 2001–2002 (NFI 2002) and 2011–2012 (NFI 2012). Detailed information about the sampling strategy of the German NFI can be found, for example, in [15] or [16]. It should be noted that the German reunification of East (new German Länder) and West Germany (old German Länder) in 1990 led to difficulties with the availability of comparable forest inventory data. The required forest conditions in the new federal states were evaluated based on forest planning data (Datenspeicher Waldfonds, DSWF) [16] representing management activities, which were practiced in their original form, until the beginning of 1993. In addition, an intermediate survey (IS 2008) on a sub-sample of the NFI plots was also carried out in 2008 (Inventurstudie 2008 und Treibhausgasinventar Wald) in order to get values for biomass carbon stocks at an additional point in time between the NFI 2002 and 2012 and with a view to open the balance for the first commitment period of the Kyoto Protocol [15, 17]. In this study the data of NFI 1987, NFI 2002, NFI 2012, DSWF and IS 2008 were therefore applied.

### Biomass

Two methods are generally used to convert field measurements of trees to above ground biomass (AB) [3]. If merchantable wood volume (volume of the stem with a diameter larger than 7 cm) of all species to a known minimum diameter is estimated, simple models have been developed to convert this to biomass using expansion factors (the ratio of total AB to merchantable wood volume) (e.g. [3, 5, 18, 19]). If, however, the forest inventory data report individual tree parameters like diameter at breast height (DBH), height, age and so on then these data can be converted to biomass directly by using biomass regression equations [3]. Germany currently applies such a single tree approach to estimate the AB using an integrated biomass function applicable to all tree dimensions developed at the FVA Baden-Württemberg. The core function of this integrated biomass function based on a modified Marklund model. It is applied for trees greater than 10 cm DBH. Also empirical data were available to fit a function for the subpopulation of trees smaller than 1.3 m height with DBH = 0. In the gap between both models a synthetic model acts as an interpolation function. The next section describes the integrated model, for more details see [20] and [21].

Trees ≥ 10 cm

1 $${\text{B}}_{\text{AB}} = {\text{b}}_{ 0} * {{\text{e}}^{\text{b}_{1}}} ^ * {}^{{\left( {{{\text{DBH}} / {\left( {{\text{DBH + k}}_{ 1} } \right)}}} \right)}} * {{\text{e}}^{\text{b}_{ 2}}} ^*{}^{{\left( {{{\text{D03}} / {\left( {{\text{D03 + k}}_{ 2} } \right)}}} \right)}} * {\text{ H}}^{\text{b}_{ 3}}$$

This biomass function sounds on empirical data between 10 cm DBH and a tree species-specific DBH threshold. Caused by the non-linear nature of the model, the risk exists to over-estimate the single tree AB in the upper extrapolation zone. To avoid or at least to reduce such effects, the last slope of the Marklund function was linearized above this tree species-specific DBH threshold using a Taylor linearization with an abortion after the first order term [20] as shown in Eq. (2):

2 $${\text{B}}_{\text{AB}} = {\text{ B}}_{\text{S}} * \, \left[ {{{ 1 { } + {\text{ b}}_{ 1} {\text{k}}_{ 1} } \mathord{\left/ {\vphantom {{ 1 { } + {\text{ b}}_{ 1} {\text{k}}_{ 1} } {\left( {{\text{DBH}}_{\text{s}} + {\text{ k}}_{ 1} } \right)^{2} }}} \right. \kern-0pt} {\left( {{\text{DBH}}_{\text{s}} + {\text{ k}}_{ 1} } \right)^{2} }}} \right]* \, \left( {{\text{DBH }}{-}{\text{ DBH}}_{\text{s}} } \right) \, + \, {{{\text{b}}_{ 2} {\text{k}}_{ 2} } \mathord{\left/ {\vphantom {{{\text{b}}_{ 2} {\text{k}}_{ 2} } {\left( {{\text{D}}_{{0 3 {\text{s}}}} + {\text{ k}}_{ 2} } \right)^{2} }}} \right. \kern-0pt} {\left( {{\text{D}}_{{0 3 {\text{s}}}} + {\text{ k}}_{ 2} } \right)^{2} }}* \, \left( {{\text{D}}_{0 3} {-}{\text{ D}}_{{0 3 {\text{s}}}} } \right) \, + \, {{{\text{b}}_{ 3} } \mathord{\left/ {\vphantom {{{\text{b}}_{ 3} } {{\text{H}}_{\text{s}} }}} \right. \kern-0pt} {{\text{H}}_{\text{s}} }}*\left( {{\text{H }}{-}{\text{ H}}_{\text{s}} } \right)$$

with B_s_ the biomass at the tree species-specific DBH threshold DBHs (Table 2). D_03s_ and H_s_ at DBH_s_ is calculated by Eqs. 3 and 4. The corresponding coefficients are listed in Table 1.

**Table 1:** Coefficients of extrapolation function 3 and 4

Tree species	Coefficients for D_03_ function		Coefficients for height function		DBH (cm)
	c_0_	c_1_	a	b	
Spruce	1.07843	0.91204	0.27407	2.22031	69.0
Pine	0.89009	0.95747	0.29722	1.98688	59.0
Beech	0.84014	0.98970	0.29397	1.76894	86.0
Oak	0.87633	0.98279	0.31567	1.63335	94.0
Soft hardwoods	0.86720	0.96154	0.28064	2.40288	113.0

3 $${\text{D}}_{{0 3 {\text{s}}}} = {\text{ D}}_{0 3} + {\text{ c}}_{0} {\text{DBH}}_{\text{s}}^{\text{c}_{1}} {-}{\text{c}}_{0} {\text{DBH}}_{\text{s}}^{\text{c}_{1}}$$

4 $${\text{H}}_{\text{s}} = {\text{ H }} + \, \left( {{\text{a }} + {\text{ b }}/{\text{ DBH}}_{\text{s}} } \right)^{ - 3} - \, \left( {{\text{a }} + {\text{ b }}/{\text{ DBH}}} \right)^{ - 3}$$

Trees ≥ 1.3 m height and <10 cm DBH

5 $${\text{B}}_{\text{AB}} = {\text{ b}}_{0} + \, \left( {\left( {\left( {{\text{b}}_{\text{s}} - {\text{b}}_{0} } \right) \, /{\text{ d}}_{\text{s}}^{ 2} } \right) \, + {\text{ b}}_{ 3} * \, \left( {{\text{DBH}} - {\text{d}}_{\text{s}} } \right)} \right) \, *{\text{ DBH}}^{ 2}$$

Trees <1.3 m height

6 $${\text{B}}_{\text{AB}} = {\text{ b}}_{0} *{\text{ H}}^{\text{b}_{ 1}}$$

Here B_AB_ is the above ground biomass (kg), DBH is the diameter at breast height (cm), b_0, 1, 2, 3, S_ and k_1, 2_ are coefficients, D_03_ is the diameter at 30 % of tree height (cm), H is the tree height (m) and d_s_ is the diameter-validity boundary (10 cm). The coefficients of the individual biomass functions are documented in the Tables 2, 3 and 4.

**Table 2:** Coefficients of biomass function for trees ≥10 cm DBH

Tree species	b_0_	b_1_	b_2_	b_3_	k_1_	k_2_	RMSE (%)
Spruce	0.75285	2.84985	6.03036	0.62188	42.0	24.0	11.2
Pine	0.33778	2.84055	6.34964	0.62755	18.0	23.0	15.6
Beech	0.16787	6.25452	6.64752	0.80745	11.0	135.0	18.8
Oak	0.09428	10.26998	8.13894	0.55845	400.0	8.0	12.1
Soft hardwoods	0.27278	4.19240	5.96298	0.81031	13.7	66.8	50.0^1^

*RMSE* root mean square error

^a^ For this function, no figure for RMSE (%) is available. Therefore the IPCC default value 50 % has been used, as described in the IPCC guidelines and good practice guidance

**Table 3:** Coefficients of biomass function for trees ≥1.3 m height and <10 cm DBH

Tree species	b_0_	b_s_	b_3_
Spruce	0.41080	26.63122	0.01370
Pine	0.41080	19.99943	0.00916
Beech	0.09644	33.22328	0.01162
Oak	0.09644	28.94782	0.01501
Soft hardwoods	0.09644	16.86101	−0.00551

**Table 4:** Coefficients of biomass function for trees <1.3 m height

Tree species	b_0_	b_1_
Spruce	0.23059	2.20101
Beech	0.04940	2.54946

The below ground biomass (BB) was also calculated on the basis of biomass functions. Here the following equation was used:

7 $${\text{B}}_{\text{BB}} = {\text{ b}}_{0} *{\text{ DBH}}^{\text{b}_{ 1}}$$

where B_BB_ is the below ground biomass (kg), DBH is the diameter at breast height (cm) and b_0_…b_1_ are coefficients. The tree species-specific coefficients, required for calculating the BB by tree-species group, can be found in Table 5.

**Table 5:** Coefficients for calculating below ground biomass

Tree species	b0	Parameter	b1	RMSE (%)	Region	Source
Spruce	0.003720	DBH (cm)	2.792465	34.6	Solling	[22]
Pine	0.006089	DBH (cm)	2.739073	26.3	Barnim	[23]
Beech	0.018256	DBH (cm)	2.321997	49.0	Solling	[22]
Oak^a^	0.028000	DBH (cm)	2.440000	50.0	Northeast France	[24]
Soft hardwoods^b^ (root biomass)	0.000010	DBH (mm)	2.529000	9.6	South Sweden	[25]
Soft hardwoods^b^ (root stump biomass)	0.000116	DBH (mm)	2.290300	15.9	South Sweden	[25]

*RMSE* root mean square error

^a^ For this function, no figure for RMSE [%] is available. Therefore the IPCC default value 50 % has been used, as described in the IPCC Guidelines and Good Practice Guidance

^b^ The mean RMSE [%] for both functions (root stump biomass + root biomass) is 24.2 %

For the conversion of tree biomass to carbon content, a value of 0.50 [26], was used [21].

### Carbon stock

On a national scale, changes in the biomass and carbon stocks of trees can be estimated based on NFI data [16]. According to IPCC [5] there are two basic methods to estimate carbon stock changes (∆C). In the gain-loss method (GLM) the ∆C are estimated by considering all relevant processes, and are calculated as the difference between carbon gains (due to growth of trees) and carbon losses (due to harvests, fires and other natural losses and disturbances) [27]:

8 $$\Delta {\text{C }} = \, \Delta {\text{C}}_{\text{G}} {-} \, \Delta {\text{C}}_{\text{L}}$$

where ΔC is the annual carbon stock change (t C a^−1^), ΔC_G_ is the annual gain of carbon (t C a^−1^) and ΔC_L_ is the annual loss of carbon (t C a^−1^). By contrast, in the stock-difference method (SDM), the ∆C are the difference of carbon stocks for a given forest area at two points of time [5, 27]:

9 $$\Delta {\text{C }} = \, {{\left( {{\text{C}}_{\text{t2}} {-}{\text{ C}}_{\text{t1}} } \right)} \mathord{\left/ {\vphantom {{\left( {{\text{C}}_{\text{t2}} {-}{\text{ C}}_{\text{t1}} } \right)} {\left( {{\text{t}}_{ 2} {-}{\text{ t}}_{ 1} } \right)}}} \right. \kern-0pt} {\left( {{\text{t}}_{ 2} {-}{\text{ t}}_{ 1} } \right)}} \,$$

where ΔC is the annual change in carbon stocks in biomass (t C a^−1^), C_t1_ is the carbon stock at time 1 (t C) and C_t2_ is the carbon stock at time 2 (t C). In Germany, the changes in biomass carbon stocks for forests remaining forests are currently calculated with the SDM using the intersection area for forest land. In general the SDM requires more data (time series of NFIs) but is considered less uncertain [5].

### Annual carbon stock change in the living biomass

**Fig. 1:**
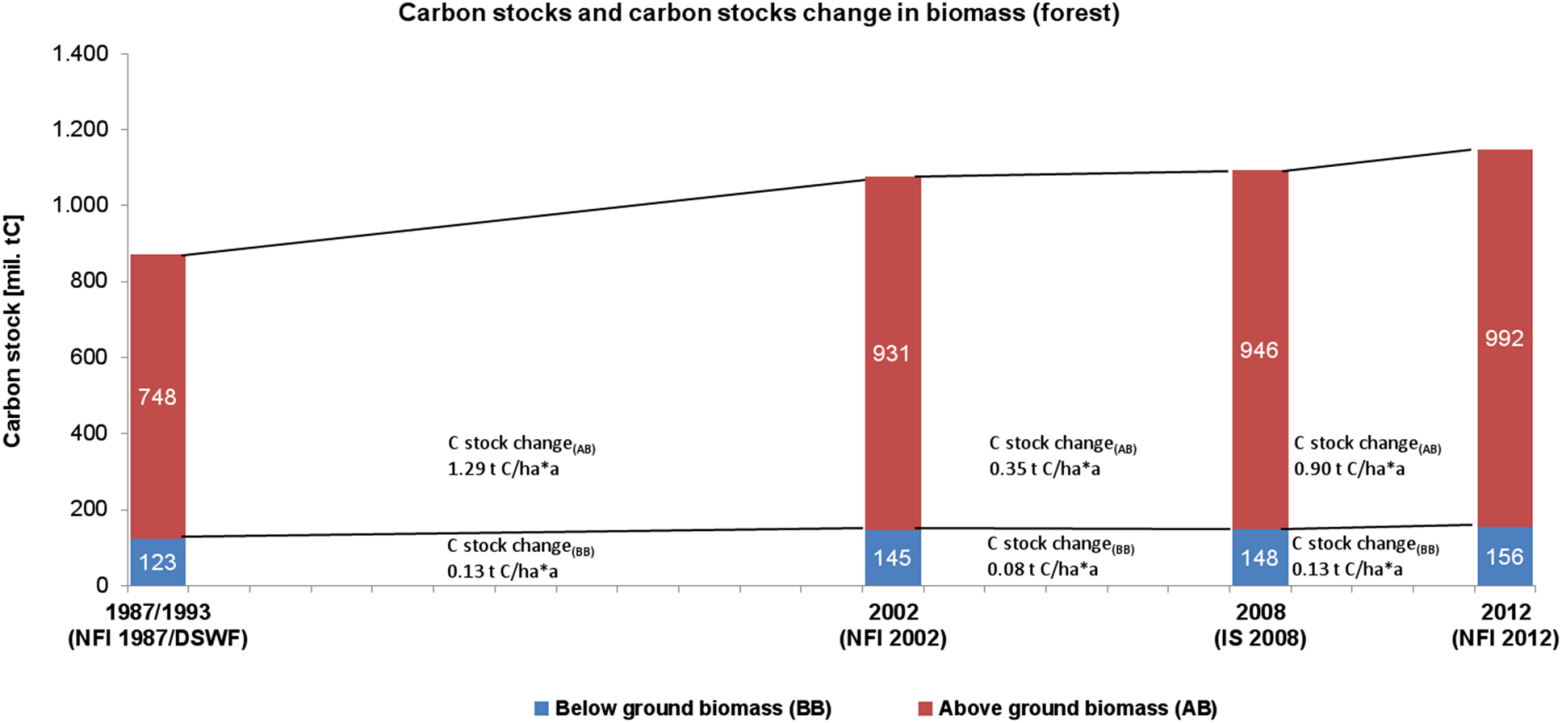
Carbon stocks and changes in forest biomass in different years (taken from [21])

With the SDM an average country specific annual emission factor (EF) (tier 2) is obtained for the time between different relevant years for which data sources are available. In our case, this has led to an EF for the period prior to 2002, expressing the average biomass change between the NFI 1987 and the NFI 2002 in the old German Länder, and between the DWSF and the NFI 2002 in the new German Länder; an EF for the period 2002 through 2008, expressing the average biomass change between the NFI 2002 and the IS 2008; and an EF for the period 2008 through 2012, expressing the average biomass change between the IS 2008 and the NFI 2012 for Germany as a whole [21]. The field measurements of the NFIs are carried out periodically, these results in “significant periodical fluctuations” (jumps) within the biomass stock changes between the individual time series (Fig. 1) but without using additional data on annual basis, no inter-annual variability can be reflected. Therefore, two different methods were developed in order to estimate annual rather than periodic fluctuations within the GHG reporting of forests in Germany.

### Application of two new emission calculation factor methods

#### The logging factor method (removal)

As calculations generally based on periodical field measurements deliver periodical average results only, the first step for improvement in order to reflect inter-annual variability is to introduce logging data, which is available annually. As logging causes losses of carbon stored in the forests biomass, it influences the change of carbon stocks towards the source direction. The higher the amount of harvested timber in one particular year compared to the periodical average, the more stock change has to be corrected towards the source direction and vice versa. This can be implemented by the logging factor method (LFM), even if no additional annual data on the opposite driver, the biomass increment, is available.

In the LFM the annual country specific emission factor (EF_LFMa_) is calculated using the following equation:

10 $${\text{EF}}_{\text{LFMa}} = {\text{ EF }}* \, ( 1+ {\text{F}}_{ 1} )$$

where EF is the periodically average country specific emission factor (t C ha^−1^ a^−1^), determined according to the given formula of IPCC, 2006 [5] (see Eq. 9), and F_1_ is the correction factor which represent the deviation of the annual fellings from the mean periodic fellings within the periods 1990–2001, 2002–2007, 2008–2012 (dimensionless), as a result of:

11 $${\text{F}}_{ 1} = {{\,\left( {{\text{L}}_{\text{fp}} - {\text{L}}_{\text{fa}} } \right)} \mathord{\left/ {\vphantom {{\,\left( {{\text{L}}_{\text{fp}} - {\text{L}}_{\text{fa}} } \right)} {{\text{L}}_{\text{fp}} }}} \right. \kern-0pt} {{\text{L}}_{\text{fp}} }}$$

where L_fa_ are the annual fellings (m^3^ under bark (m^3^ u. b.), i.e. without bark and cutting losses deducted) and L_fp_ are the mean periodic fellings within the periods 1990–2001, 2002–2007, 2008–2012 (m^3^ u. b.). For this, the annual reported logging data of the Food and Agriculture Organization (FAO) were used.

### The growth factor method (gain and removal)

Within the growth factor method (GFM) the calculations of the annual changes in stocks (∆C_T_) (EF in the narrow sense) are performed in the form of a balance, which is equal to the equation of the GLM (Eq. 8):

12 $$\Delta {\text{C}}_{\text{T}} = \Delta {\text{C}}_{\text{G}} {-}\Delta {\text{C}}_{{{\text{L}}({\text{T}})}}$$

where ∆C_G_ is the average annual gross increment of carbon in the respective period (in the narrow sense the annual carbon gain) (t C ha^−1^ a^−1^), calculated by using Eq. (15), and ΔC_L(T)_ is the annual loss of carbon due to fellings (t C ha^−1^ a^−1^), resulting from:

13 $$\Delta {\text{C}}_{{{\text{L}}({\text{T}})}} = {\text{ L}}_{\text{fa}} *{\text{ F}}_{ 3} *{\text{ BD }}*{\text{ CF }}*{\text{ F}}_{ 1} *{\text{ F}}_{ 2} /{\text{a}}$$

In this case, L_fa_ are the annual fellings (m^3^ over bark (m^3^ o. b.), i.e. merchantable wood volume), taken from the FAO database FAOSTAT (Food and Agriculture Organization Corporate Statistical Database), F_3_ is the factor for the conversion of m^3^ u. b. to m^3^ o. b. (dimensionless) taken from the result database of the NFI 2012 (http://www.bwi.info), BD is the basic density (t m^−3^) calculated as weighted mean for the NFI main species oak, beech, spruce and pine using values taken from the IPCC Guidelines (2006) [5], CF is the factor for the conversion of tree biomass to carbon biomass (dimensionless), F_1_ is the correction factor which represent the deviation of the annual fellings from the mean periodic fellings within the periods 1990–2001, 2002–2007, 2008–2012 (dimensionless) and a is the forest area (ha).

As the periodic mean of felling data (L_fp_) extracted from FAOSTAT deviate from the periodic felling data (F_fpNFI_) provided by the NFI an average periodic correction factor (F_2_) was formed using Eq. (14) resulting in 1.05 for the period between the NFI 2002 and the IS 2008, 0.99 for the time between IS 2008 and NFI 2012 and 1.03 for the period from 1990 to 2001:

14 $${\text{F}}_{ 2} = \, {{{\text{L}}_{\text{fpNFI}} } \mathord{\left/ {\vphantom {{{\text{L}}_{\text{fpNFI}} } {{\text{L}}_{\text{fp}} }}} \right. \kern-0pt} {{\text{L}}_{\text{fp}} }}$$

The gross increment is defined as the growth including removals (hereinafter referred to as increment) [28]. Generally it should also include mortality, but as there is no explicit data on this available and losses due to mortality in Germany are very small, they can not be taken into account at this stage and are neglected. So the calculation of the average annual gross increment of carbon (∆C_G_) was carried out by using the following equation:

15 $$\Delta {\text{C}}_{\text{G}} = \,\,{{\left( {{\text{C}}_{\text{t2}} {-}{\text{ C}}_{\text{t1}} + \varSigma \Delta {\text{C}}_{{{\text{L}}\left( {\text{T}} \right)({\text{t2}} - {\text{t1}})}} } \right)} \mathord{\left/ {\vphantom {{\left( {{\text{C}}_{\text{t2}} {-}{\text{ C}}_{\text{t1}} + \varSigma \Delta {\text{C}}_{{{\text{L}}\left( {\text{T}} \right)({\text{t2}} - {\text{t1}})}} } \right)} {{\text{ t}}_{ 2} {-}{\text{ t}}_{ 1} }}} \right. \kern-0pt} {{\text{ t}}_{ 2} {-}{\text{ t}}_{ 1} }}$$

where C_t1_ is the total biomass in carbon at time 1 (t C), C_t2_ is the total biomass in carbon at time 2 (t C) and ΣΔC_L(T)(t2–t1)_ is the sum of the annual fellings within the corresponding period (t C ha^−1^).

## Results

### Total biomass carbon stocks in German forests

**Fig. 2:**
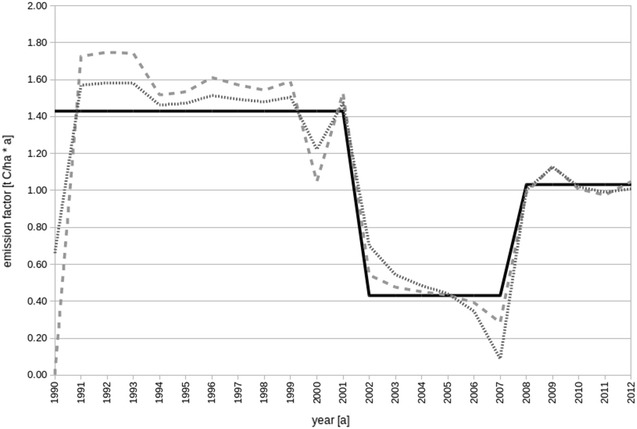
Comparison within the annual fluctuations of the emission factors. Logging factor method (*light* *gray broken line*), growth factor method (*dark gray dotted line*), periodic emission factors (*black solid line*) as indicated in the national greenhouse gas inventory reports of Germany (NIR 2010–2015)

As specified in Table 6, the total biomass carbon stock of the old German Länder was calculated with 92.26 t C ha^−1^ in NFI 1987 and 111.20 t C ha^−1^ in NFI 2002. This corresponds to a ∆C of 1.26 t C ha^−1^ a^−1^ between the two national forest inventories. Comparing the total biomass carbon stocks of the new German Länder with 67.80 t C ha^−1^ in 1993 (DSWF) and 84.24 t C ha^−1^ in NFI 2002, the ∆C results in a value of 1.83 t C ha^−1^ a^−1^. After reunification in 1990 until 2002, the ∆C for the whole German forests was calculated as an area-weighted average with 1.43 t C ha^−1^ a^−1^ (Fig. 2; Table 6). Whereas the ∆C between the NFI 2002 and the IS 2008 was calculated with 0.43 t C ha^−1^ a^−1^ (Figs. 1, 2; Table 6) as reference to the total biomass carbon stocks of 2002 with 103.36 t C ha^−1^ and 2008 with 105.97 t C ha^−1^. The ∆C determined from the total biomass carbon stocks of IS 2008 (106.14 t C ha^−1^) and NFI 2012 (110.28 t C ha^−1^), however, was 1.03 t C ha^−1^ a^−1^ (Table 6; Fig. 2).

**Table 6:** Biomass carbon stocks and changes within subsequent periods, based on the German National Forest Inventories

Period	Year (a)	Forest area (ha)	C_AB_ (t C ha^−1^)	C_BB_ (t C ha^−1^)	C_TB_ (t C ha^−1^)	EF (∆C) (t C ha^−1^ a^−1^)
1987–2002	1987	7,348,890.12	80.20	12.06	92.26	1.26^a^
	2002		96.71	14.50	111.20	
1993–2002	1993	2,852,457.00	55.66	12.15	67.80	1.83^b^
	2002		71.74	12.49	84.24	
2002–2008	2002	10,368,393.65	89.49	13.87	103.36	0.43
	2008		91.59	14.38	105.97	
2008–2012	2008	10,306,813.31	91.74	14.40	106.14	1.03
	2012		95.35	14.93	110.28	

*C*_*AB*_  carbon in above ground biomass

*C*_*BB*_  carbon in below ground biomass

*C*_*TB*_  carbon in total biomass (C_TB_ = C_AB_ + C_BB_)

*EF*  emission factor

*∆C*  annual biomass carbon stock change

^a^ Old German Länder

^b^ New German Länder

### Annual biomass carbon stock change

Figure 2 shows the annual fluctuations between the individual carbon stock changes (referred to here as EF) calculated on the basis of the LFM and the GFM, in comparison to the revised EFs indicated in the German national greenhouse gas inventory reports (NIR) for the periods 1990–2001, 2002–2007 and 2008–2012 (Tables 6, 7).

**Table 7:** Comparison of calculated emission factors

Year (a)	EF_NIR_ (t C ha^−1^ a^−1^)	EF_LFM_ (t C ha^−1^ a^−1^)	EF_GFM_ (t C ha^−1^ a^−1^)
1990	1.43	0.00	0.66
1991	1.43	1.73	1.57
1992	1.43	1.75	1.58
1993	1.43	1.74	1.58
1994	1.43	1.52	1.46
1995	1.43	1.53	1.47
1996	1.43	1.61	1.51
1997	1.43	1.57	1.49
1998	1.43	1.54	1.48
1999	1.43	1.59	1.50
2000	1.43	1.05	1.22
2001	1.43	1.53	1.47
2002	0.43	0.54	0.70
2003	0.43	0.48	0.54
2004	0.43	0.45	0.48
2005	0.43	0.43	0.44
2006	0.43	0.39	0.35
2007	0.43	0.28	0.09
2008	1.03	0.99	1.00
2009	1.03	1.13	1.13
2010	1.03	1.01	1.02
2011	1.03	0.97	0.99
2012	1.03	1.05	1.01

*EF*_*NIR*_  emission factor as indicated in the national greenhouse gas inventory reports of Germany

*EF*_*LFM*_  emission factor calculated on the basis of the logging factor method

*EF*_*GFM*_  emission factor calculated on the basis of the growth factor method

The following results can be determined: (1) In the period 2008–2012, the sequences of the individual EFs, within the different calculation methods are similar (Fig. 2), due to the fact that the annual wood harvests of the forest management are at about the same level (Table 8). Here, the adapted logging values ranges between 47,755,606 m^3^ u. b. (2009) and 55,770,598 m^3^ u. b. (2011) (Table 8), which results in minimum and maximum EFs of 0.97 t C ha^−1^ a^−1^ (2011) and 1.13 t C ha^−1^ a^−1^ (2009) (Fig. 2; Table 7). (2) Larger differences between the EFs were found in the periods 1990–2001 and 2002–2007 (Table 7). These annual fluctuations are caused in changes of tree growth and harvesting, as explained in more detail below.

From 1990 till 2007 the range of the EFs, calculated according to the LFM, was between 0.00 t C ha^−1^ a^−1^ (1990) and 1.75 t C ha^−1^ a^−1^ (1992), whereas, the lowest EF within the GFM, was 0.09 t C ha^−1^ a^−1^ (2007) and the highest EF was reached with 1.58 t C ha^−1^ a^−1^ (1992, 1993). The adapted loggings are indicated with values of 33,942,620 m^3^ u. b. (1992) up to 87,248,210 m^3^ u. b. (1990) (Table 8).

**Table 8:** Logging values, FOASTAT (L_FAO_) vs. calculated using the conversion factor F2 (L_F2_)

Year (a)	L_FAO_ (m^3^ u. b.)	L_F2_ (m^3^ u. b.)
1990	84,707,000	87,248,210
1991	33,618,000	34,626,540
1992	32,954,000	33,942,620
1993	33,152,000	34,146,560
1994	39,813,000	41,007,390
1995	39,343,000	40,523,290
1996	37,014,000	38,124,420
1997	38,207,000	39,353,210
1998	39,052,000	40,223,560
1999	37,634,000	38,763,020
2000	53,710,000	55,321,300
2001	39,483,000	40,667,490
2002	42,380,000	44,596,823
2003	51,182,000	53,859,240
2004	54,504,000	57,355,008
2005	56,946,000	59,924,744
2006	62,290,000	65,548,280
2007	76,728,000	80,741,506
2008	55,367,000	55,001,141
2009	48,073,268	47,755,606
2010	54,418,357	54,058,767
2011	56,141,575	55,770,598
2012	52,338,132	51,992,288

As illustrated in Fig. 2, large fluctuations within the annual EFs are linked to years of extreme weather events. In our case, these are the winter storms “Vivian” and “Wiebke” (1990), “Lothar” (1999) and “Kyrill” (2007), which are responsible for large amounts of wind throw timber, reported with 763,680,000 m^3^ in 1990, 33,890,000 m^3^ in 1999 [29] and about 37,000,000 m^3^ in 2007 [30]. Consequently, in the years 1990, 2000 and 2007 the removals of timber were much larger than foreseen in the forest year concerned. If the tree growth is exceeded by timber removals, a reduction of carbon stocks within the forest stands is considered (Fig. 2).

In particular, the fluctuations of the annual EFs between the two methods reflect the application of different parameters within the calculation methodologies. Compared to the LFM, the annual EFs of the GFM are calculated with the average annual gross increment of carbon in the respective period (∆C_G_)_,_ and additionally with the EF of the annual fellings (∆C_L(T)_) (see Eq. 12). Here, in chronological order, the ∆C_G_ was calculated with 2.20 t C ha^−1^ a^−1^ in 1990–2001, as weighted mean of the ∆C_G_s of the old German Länder (2.12 t C ha^−1^ a^−1^) and the new German Länder (2.28 t C ha^−1^ a^−1^), 1.46 t C ha^−1^ a^−1^ in 2002–2007 and 1.94 t C ha^−1^ a^−1^ in 2008–2012. Between the different periods, it has been observed that the average EFs of the GFM increases with increasing ∆C_G_s_._ Considering, however, the time series 1990–2012 of the LFM, neglecting the influence of ∆C_G_, a nearly constant mean relative deviation of the respective annual EFs from the mean periodic EFs (Table 6) can be observed, here especially for the first two periods 1990–2001 and 2002–2007, as shown in Fig. 2. Whereas, the mean relative deviation of the annual GFM EFs to the mean periodic EFs (Table 6) is greater in the period 2002–2007, than in the period from 1990 to 2001 (Fig. 2).

## Discussion

### Method for calculating forest carbon balance based on forest inventory data

It is known, that the annual forest biomass carbon balance can be derived from forest inventories as (1) estimations of changes in carbon stocks (SDM) or (2) from the annual balance between estimated gains and losses of carbon (GLM). The methods differ in the fact, that the uncertainties in the GLM are dominated by model errors due to the different components which only partly are usually derived from statistical forest inventory data, whereas the uncertainties in the SDM, which are derived from net-carbon changes based on repeated statistical forest inventories, is dominated by the sampling error, especially in cases where the net-carbon changes are very small.

The methodological guidance provided by the IPCC states that uncertainty estimates must accompany the annual estimates of GHG emissions. Various uncertainties have to be taken into account in calculation of carbon stocks [21]. In the process, as seen in, for example [19, 31] and [32], a large number of predictors used in the LULUCF sector reporting comes from design-based NFIs replaced in time [33] or from supplementary design-based probability samples. This corresponds to the current studies of [33], which summarized, that the magnitude of reported relative errors depends on the sampling design and the uncertainty in applied models.

However, some of the uncertainty in the estimation of the annual EFs of the living AB arises because biomass cannot be directly measured. A number of error sources (e.g. errors in the biomass functions or in the carbon conversion factor) enter into the process of deriving forest biomass and carbon stocks, and of deriving their changes [21]. Furthermore, the quality of logging-statistics data is poor, since many subsets of the data are based on expert assessments [21, 34]. Comparisons between the annual reported logging data of the German wood balances (Thünen-Institute, Institute of International Forestry and Forest Economics) and the annual reported logging data taken from the FAO database FAOSTAT for the time series 1990-2012, however, are matching from 1995 onwards. For this reason, the logging statistics can be used as a data source for the calculation of the annual EF in this article.

The application of the SDM with the use of periodic data delivers average periodic emission factors only. These data are of high statistical quality (low statistical uncertainties, high precision) for the periods. However they do not reflect short term variations and their use for all single years in the periods can be considered as insufficient in terms of time series with annual values. With the use of additional data like harvest statistics and data on growth and the application of the LFM or GFM a methodological improvement is available. With this improvement it is possible to overcome the previously mentioned limitation of the SDM currently in use and better reflect the inter-annual changes in the emission time series. At the same time, the use of harvest rates and/or increments as additional parameters, introduces further sources of uncertainty in addition to e.g. inter-annual variation, thus increasing the overall uncertainty of the results. Since the available datasets are based on limited surveys, the uncertainty of the available harvest data is not provided. Therefore, the extent of the additional uncertainty due to the application of the LFM or GFM cannot be quantified.

### Annual variability in forest carbon balance

The trajectory of the emissions and removals in time (period as well as annual estimations) is basically composed by the biomass gains, reducing the emissions, but increasing the EF for the biomass pool, and the biomass losses, increasing the emissions, but decreasing the EF for the biomass pool, as described above. Thus gains (increment, growth) and losses (harvest, logging, etc.) influence the modulation in opposite ways [35] and the relation between their absolute values determines the absolute amount of changes in carbon stocks.

With the application of the above described methods it can be found that the level of emissions (EF) influences the level of modulation around the average EF (as estimated without adjustments as the periodic average, black solid line Fig. 2). Thus in the period prior to 2002, the effect of the modulation is larger than in the period between 2002 and 2008. This supports the above mentioned statement, and suggests that in this case, the gains have a higher significance for the intensity of the modulation (since the pool is a net sink) than the losses (fellings, disturbance, etc.).

Obviously the gains and losses interact in a certain way. Basics of this interaction are known and well understood (see above, Fig. 2, or [36]). The more detailed analysis of circumstances influencing the application of the here presented annual methods to the already in use periodic method is a further field of research.

The average periodic values of the increment are more variable then the average periodic values of the harvest rates. This leads to a larger relative modulation of the EF in the period 2002–2008 than in the period 1990–2001 when the GFM is applied compared to the use of the LFM only.

Both methods can be used to reflect the inter-annual fluctuations in the time series for emissions within the periods between the NFI inventory cycles. LFM introduces harvest rates time series only as additional parameter beyond the parameters currently used by the SDM and therefore is easier to implement. The other (GFM) additionally uses information about changing increment over time. The results of the current application of both proof that the resulting time series trajectories are comparable and matching the data available on periodical basis like intended by the design of the methods. Considering current information on increment data is only available as periodical data, the influences of inter-annual variation of increment cannot yet fully be determined. To take these fluctuation fully into account, compatible annual time series on increment are necessary, which might be subject to further research.

As described, the application of the pure SDM only allows for the statistical quantification of uncertainties. As the uncertainties of the newly introduced datasets are partially or fully unknown, statistical uncertainties for the annual EFs resulting from the application of the here described methods cannot be mathematically derived. Nevertheless, the uncertainty calculations regarding the underlying periodical data can still be provided and used as rough indicator for the quality of the resulting time series.

## Conclusion

The application of the methods presented in this study resulting in more differentiated time series of emissions, increases plausibility of the provided time series for the German emissions reporting, and also the comparability with neighboring countries like Switzerland or Austria where similar approaches are used to derive emission time series.

Currently the logging factor is available as annual value, the growth factor is actually only a periodic value. Thus the former leads to a reflection of interannual variability of the emission factor, while the latter does only influence the periodic data. As a further improvement the concept in general could also be extended and the growth factor may also be turned into an annual value. Therefore, further annual datasets like climatic parameters may be used to modulate this factor over time. As the impact of such values is generally known, but not yet quantified in the context of emission reporting for Germany, this would be subject of further research. It is intended to take this approach in the German greenhouse gas reporting in order to meet the request for annually adjusted values.

As NFIs are carried out periodically, the EFs in the GHG inventory have to be extrapolated until the next NFI cycle becomes available. Even as the time series of the EFs have to be recalculated after each NFI cycle, the application of the suggested method on these extrapolations allows to provide more realistic emission data on short term basis and therefore to improve the knowledge about emission trends. This can be a benefit also for the monitoring of emission reduction policies and measures.

## Abbreviations

AFOLU: agriculture, forestry and other land-use
AB: above ground biomass
BB: below ground biomass
BWI: Bundeswaldinventur
CO_2_: carbon dioxide
COP: conference of the parties
ΔC: annual carbon stock change
DBH: diameter at breast height
DSWF: Datenspeicher Waldfonds
EF: emission factor
FAO: Food and Agriculture Organization of the United Nations
FCCC: Framework Convention on Climate Change
FVA: Forstliche Versuchs- und Forschungsanstalt Baden-Württemberg
GFM: growth factor method
GHG: greenhouse gases
GLM: gain loss method
GPG: good practice guidance
IPCC: Intergovernmental Panel on Climate Change
IS 2008: Inventurstudie 2008
LFM: logging factor method
LULUCF: land use, land-use change and forestry
NFI: National Forest Inventory
NIR: National Inventory Report
UNFCCC: United Nations Framework Convention on Climate Change
RMSE: root mean square error
SDM: stock-difference method
